# A flexible pressure sensor with highly customizable sensitivity and linearity via positive design of microhierarchical structures with a hyperelastic model

**DOI:** 10.1038/s41378-022-00477-w

**Published:** 2023-01-04

**Authors:** Zhenjin Xu, Dezhi Wu, Zhiwen Chen, Zhongbao Wang, Cong Cao, Xiangyu Shao, Gang Zhou, Shaohua Zhang, Lingyun Wang, Daoheng Sun

**Affiliations:** 1 Department of Mechanical and Electrical Engineering, Xiamen University, Xiamen, 361005 China; 2 Shenzhen Research Institute of Xiamen University, Shenzhen, 518057 China; 3 Beijing Key Laboratory of Long-Life Technology of Precise Rotation and Transmission Mechanisms, Beijing Institute of Control Engineering, Beijing, 100094 China

**Keywords:** Electrical and electronic engineering, Biosensors

## Abstract

The tactile pressure sensor is of great significance in flexible electronics, but sensitivity customization over the required working range with high linearity still remains a critical challenge. Despite numerous efforts to achieve high sensitivity and a wide working range, most sensitive microstructures tend to be obtained only by inverting naturally existing templates without rational design based on fundamental contact principles or models for piezoresistive pressure sensors. Here, a positive design strategy with a hyperelastic model and a Hertzian contact model for comparison was proposed to develop a flexible pressure sensor with highly customizable linear sensitivity and linearity, in which the microstructure distribution was precalculated according to the desired requirement prior to fabrication. As a proof of concept, three flexible pressure sensors exhibited sensitivities of 0.7, 1.0, and 1.3 kPa^−^^1^ over a linear region of up to 200 kPa, with a low sensitivity error (<5%) and high linearity (~0.99), as expected. Based on the superior electromechanical performance of these sensors, potential applications in physiological signal recognition are demonstrated as well, and such a strategy could shed more light on demand-oriented scenarios, including designable working ranges and linear sensitivity for next-generation wearable devices.

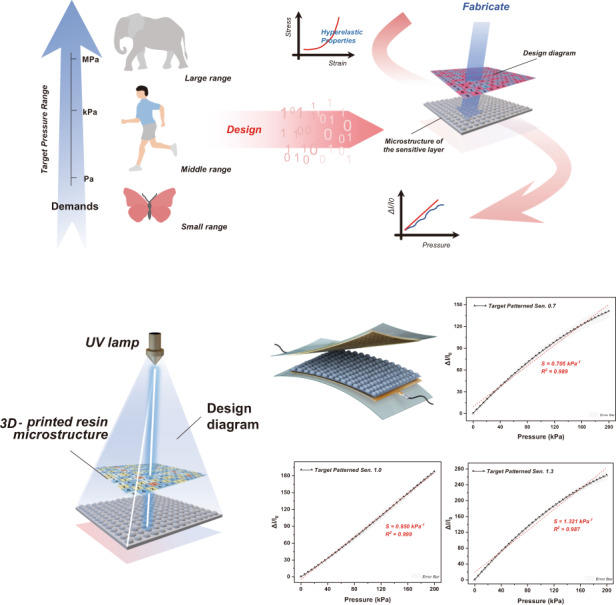

## Introduction

Structural modulation in micron-sized patterns within active layers is considered a promising approach for enhancing the performance of flexible pressure sensors, thereby expanding the targeted applications to electronic skin^1–4^, healthcare monitoring^5–7^, and human‒machine interfaces^8–10^. High sensor performance relies on employing new microengineering strategies in terms of geometric and spatial designs. For instance, enhanced sensitivity and response speed can be achieved via the controllable introduction of microstructures with highly regular shapes, such as pyramids^11–13^ or microdomes^14–16^, by increasing the compressibility and reducing the viscoelasticity of hyperelastic elastomers in a modulus-tunable manner^17,18^. However, a significant challenge remains as the external pressure increases: the sensors inevitably suffer from deformation saturation due to the elastomer’s compressibility reduction^19^, which leads to sensing limitations in a high-pressure regime.

The passive design of irregular microstructures with multiscale hierarchical properties offers an optimal solution for realizing continuous deformation with pressure^20,21^. Previously, methods of inverting naturally occurring microstructural templates (e.g., pollen grains^22^ or petals^23^, human skin^24,25^, abrasive paper^26^, and kirigami patterns^27^) or MEMS-fabricated artificial patterns^28–31^ have been introduced for piezoresistive sensors to fabricate active layers. For example, Geng et al. reported an ordered multilevel microstructure and explored the regulations of its radius and spatial distribution impacting the sensor performance merely via simulations and experiments^32^, rather than from a quantitative perspective. With this in mind, such passive strategies still lack clarity regarding rational design based on fundamental contact principles or models^33–37^, resulting in limitedly targeted performance implementation. To this end, tailoring microstructures in a positive rather than passive fashion is promising for eliminating such restrictive limitations fundamentally based on appropriate theoretical calculations to further determine the morphological and spatial parameters.

Although some forward-looking approaches have emerged based on proactive strategies, there remains an important concern for positive design in micropatterned devices, i.e., the constitutive model mismatch for active layer deformation. Several reported examples of elucidating the principle of resistance variation with pressure have employed Hertz contact theory, attempting to establish a relationship between contact area change and sensitivity, but only qualitative analysis has been used^5,32,38^. Zhou’s group proposed a rational assignment method of the gradient microdome architecture for proactive performance improvement on the basis of Hertzian contact, including the target designs of both the size and the count^38^. Nevertheless, such positive schemes are unable to ensure highly predictable performance since they are inappropriate for describing a hyperelastic material utilizing elastic solid contact theory for large deformations^39,40^.

Here, we propose a new positive design strategy of microhierarchical structures for addressing the abovementioned concerns based on the hyperelastic mechanics of sensitive elastomers. Unlike the simple application of Hertzian contact on a hyperelastic material, which may cause an elastic contact shift (Text S3), our modified contact theoretical model could predict more accurate deformation parameters by referring to the corresponding finite element analysis (FEA) simulation, thus achieving the targeted designs and implementations for microengineered sensors. As a proof of concept, as-fabricated pressure sensors featuring highly customizable sensitivity (0.7, 1.0, and 1.3 kPa^−1^) and high linearity (*R*^2^ ≈ 0.99) over a predesigned linear working range (approximately 200 kPa) have been developed, particularly on obviously antisaturated compensation at the predetermined pressure. The prototype sensor also presented a fast response/release time of 12.5/37.5 ms, a tiny limit of detection (LOD) of 35 Pa, and good repeatability for more than 10,000 cycles of repeated loading/unloading. This straightforward, positive design philosophy could allow such demand-oriented scenarios, including a designable working range and linear sensitivity, to be successful.

## Results and discussion

### Design concept and sensing mechanism of the sensitivity-customized sensor

**Fig. 1:**
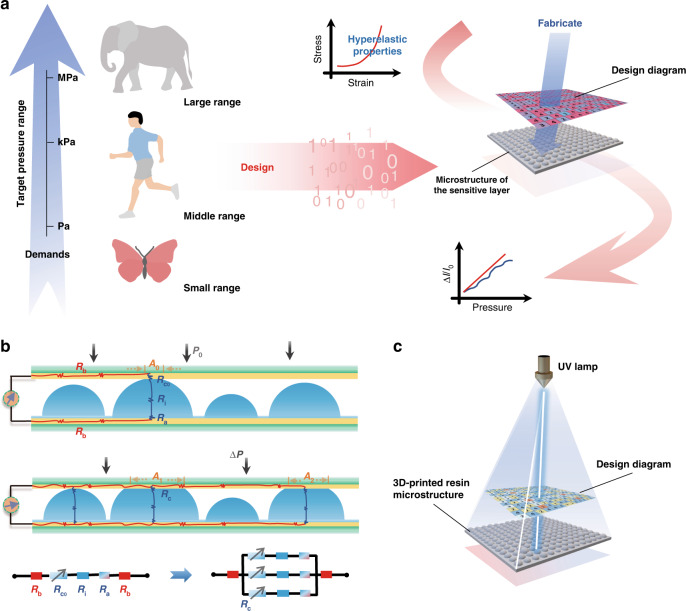
**a** Design concept of the developed sensitivity-customized sensor. **b** Sensing mechanism of the microhierarchical structure-based sensor. **c** The applied PμSL-based 3D printing technology for realizing the positive design of the multistage protrusion with a high precision of 10 μm

Matching between practical sensing resolution and an appropriate pressure range according to actual demands can be considered a feasible course to make flexible sensors more suitable for certain applications since a scenario may emphasize the macroscale working range and de-emphasize much higher sensitivity, such as tire tread stability testing^41^, and other scenarios may be quite the opposite, such as pulse waveform recognition^42^. Our positive design strategy for scenario-specific pressure sensors enables the achievement of highly customizable sensitivity and linearity (Fig. 1a). For the initial design step, motivated by the actual requirement of multiscale applications for different target pressure ranges, here, we realize both the corresponding sensitivity and linear working range via a positive design scheme, which is based on the hyperelastic properties of sensitive materials to predict real-time deformation parameters more efficiently and accurately. By applying our modified hyperelastic contact model, we can directly autocompute the specific size and number of microdome pixels at each stage in hierarchical structures. For the fabrication, the design diagram presented here provides the following 3D printing template for the microstructures of the sensitive layer, featuring antisaturation characteristics over the full-scale pressure range (Fig. S1).

To further demonstrate the positive design concept, Fig. 1b illustrates the sensing mechanism of the predesigned sensitivity-customized sensor, which mainly perceives external stimuli through the change in contact resistance. Briefly, as the sensor is subjected to pressure, the microstructure of the conductive sensitive layer is compressed and deformed to increase the contact area with the upper electrode, and then the contact resistance is reduced. The initial resistance of the uncompressed sensor can be calculated by

1 $${{{\mathrm{R}}}} = R_a + R_b + R_i + R_{{{\mathrm{c}}}}$$

where $R_a$, $R_b$, and $R_i$ represent the contact resistance between the bottom of the sensitive layer and electrodes, the resistance of the electrodes and the bulk resistance of the sensitive layer, respectively. By applying a certain load to the upper electrodes, the contact resistance between the upper electrodes and sensitive layer, which is defined as $R_{{{\mathrm{c}}}}$, becomes crucial to the sensing sensitivity, while the above resistances remain nearly constant and are considerably smaller than $R_{{{\mathrm{c}}}}$, which means they can be ignored^28^. By rewriting the sensitivity in terms of $R_{{{\mathrm{c}}}}$ and the pressure change $\Delta {{{\mathrm{P}}}}$, the sensitivity can be expressed as

2 $${{{S}}} = \frac{{\Delta {{{I/}}}I_0}}{{\Delta {{{P}}}}} = \frac{{\left( {\frac{U}{{R_c}} - \frac{U}{{R_{c0}}}} \right){{{\mathrm{/}}}}\frac{U}{{R_{c0}}}}}{{\Delta {{{P}}}}} = \frac{{\frac{{R_{c0}}}{{R_c}} - 1}}{{\Delta {{{P}}}}} = \frac{{\frac{A}{{A_0}} - 1}}{{\Delta {{{P}}}}}$$

where $A_0$ and $A$ are the initial and compressed contact areas, respectively.

Therefore, the sensitivity is positively correlated with the contact area of microstructures as external pressure is applied. Overall, regulating the linear change in contact area as pressure increases is the key to realizing the sensitivity design over a linear working range. In this work, we employed PμSL-based 3D printing technology (Fig. 1c) to realize the positive design of multistage protrusions with a precision of approximately 10 μm to ensure customized desirable sensitivity.

### Hyperelastic contact theoretical model

As elucidated in Section “Design concept and sensing mechanism of the sensitivity-customized sensor”, it is believed that the sensing sensitivity is linearly related to the rate of increase of the contact area, so maintaining the contact ratio within a certain working range allows the realization of a positive design for desirable sensitivity under externally applied pressure. By revealing the relationship among the contact area, compression height, and pressure of hemispheric pixels, the parameters of multistage dome-like microstructures that meet the requirements of high linearity can be well designed.

To make the isotropic elastic properties of our hyperelastic elastomer more appropriate for characterizing flexible pressure sensing systems, we employed the neo-Hooken model, which is reduced from the best-known strain-energy function formulation, the Moony-Rivlin model for simplicity^43^. Taking inspiration from the module measurement of the sphere squeezing case in Claude’s^44^ and Karssemeijer’s^45^ work, we determined the two major parameters for properly reflecting the large deformation based on hyperelastic mechanics, i.e., the compression height $\Delta h$ and contact radius $R_c$, such that

3 $$\Delta h = \left( {1 - \beta } \right) \cdot R^ \ast$$

4 $$R_c = R^{^ \ast } \times \sqrt {\frac{{1 - \emptyset _c^2}}{{\alpha _c}}}$$

where *β* and R^*^ are the compression ratios of the overall height and initial radius of the hemisphere pixel, respectively, and ∅_*c*_ and *α*_*c*_ represent the nominal height and tangentially principal extension ratio for the contact surface, respectively.

Although we concentrate on the critical variables (Δ*h* and *R*_*c*_) in this compression-dependent behavior, which then determine the current contact area and the shape configuration of hierarchical structures, there are two factors related to the value range determination that should also be noted: neither the nominal height φ nor the extension ratio α can be assigned to zero. If we assume that φ converges to zero at the pole of the hemisphere-shaped structure and that an extensive condition appears at this point only, i.e., remains unchanged, Eqs. 3 and 4 become unsolvable. Naturally, introducing the preloading pressure to avoid this potential problem allows these variables to be greater than zero, and is also consistent with the practical experiments since the final sensor encapsulation will apply an initial prestress on the sensitive layers. The introduction of our preloading procedures is described in Section “Prototype sensor test”, and the deprivation process for these key parameters is detailed in Text S1.

Furthermore, to design the microstructured array for preferred sensitivity in versatile scenarios, an approach to determine microhierarchical structures has also been proposed. In brief, we have attempted to build a dynamic equilibrium relationship between the total force that a single-staged array should withstand and the actual force that has been applied. Thus, since the sensitivity is determined by the change in the contact area, for a positive design, we can define

5 $$\frac{{S_n}}{{S_1}} = 1 + (n - 1) \cdot k(n \ge 2)$$

where *k* is the desired sensitivity for *n*-level hierarchical microstructures and *S*_*n*_ and *S*_1_ represent the contact area between two adjacent stages on the head and end levels, respectively. The total force $F_n$ on the *n*th-staged pixels when in contact can be described by the following equation:

6 $$F_{n} = n \cdot \frac{\rm{WR \times SA}}{\rm{TO}}$$

where WR, SA, and TO indicate the working range, sensing area, and total hierarchical orders of the predesigned sensor, respectively. According to the balance between $F_n$ on each stage of the hemispherical microstructure and its actual applied force, it can also be expressed as:

7 $$F_n = \mathop {\sum }\limits_{i = 1}^n m_i \cdot F_{n \to i}$$

where *m* represents the number of micro pixels in the current stage and $F_{n \to i}$ is the component force of each pixel. Similarly, the total contact area can be defined as

8 $$S_n = \mathop {\sum }\limits_{i = 1}^n m_i \cdot \pi R_{ci}^2$$

For the compression height of array conditions, we can determine the deformation of the microhemispheres under current pressure, which is

9 $$\Delta h_n = \left( {1 - \beta _n} \right) \cdot R^ \ast _n = \left( {1 - \beta _{n - 1}} \right) \cdot R^ \ast _{n - 1} - \Delta h_{n - 1} = \ldots = \left( {1 - \beta _1} \right) \cdot R_{\,\,\,1}^{\ast } - \mathop {\sum }\nolimits_{i = 1}^{n - 1} \Delta h_i$$

With this in mind, the (*n* − 1)^th^-staged microstructure radius is determined, and then the *n*^th^-staged radius can be further calculated as

10 $$R_{n - 1}^ \ast = R_n^ \ast - \Delta h_n$$

Thus, the target parameters (i.e., *m*_*n*_ and *R*_*n*_) can be obtained by solving the above equations simultaneously.

### Fabrication of the microhierarchical structures

**Fig. 2:**
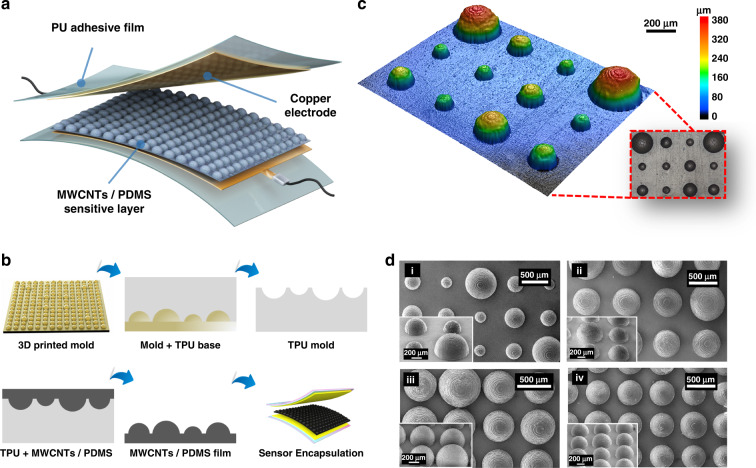
**a** Schematic diagram of the sensor with the MWCNT/PDMS sensitive film. **b** Fabrication process of the flexible pressure sensor. **c** 3D surface scanning image of the multistage protrusion. **d** SEM images of the sensitivity-customized layer with different sensitivities from the top view and the insets from the side view: (i) target sensitivity (Sen.) of 0.7 kPa^−^^1^ based on Hertzian contact, (ii) target Sen. of 0.7, (iii) 1.0, and (iv) 1.3 kPa^−^^1^ based on hyperelastic contact

To further evaluate our hyperelasticity-based modified model for a proof of concept, we designed certain flexible pressure sensors in a predetermined linear region. A schematic of the sensor structure is shown in Fig. 2a, and the corresponding preparation mainly involved three steps, as depicted in Fig. 2b: the design calculation for the size and number of hierarchical pixels during each stage and its corresponding layout, the fabrication of a structured template and sensitive layer, and the final sensor encapsulation. In Step I, we first selected a proper conductive elastomer to serve as a sensitive substrate, that is, a multiwalled carbon nanotube and polydimethylsiloxane (MWCNT/PDMS) conductive composite thin film. After the stress‒strain curve was plotted, we determined the hyperelastic material constant for further application to our modified contact model. Second, based on the actual requirement of a specific application, the desired working range and perceiving resolution (e.g., every 10 kPa or 1 kPa) were determined, and thus the order and number of hierarchical microstructures were calculated. By achieving a rational distribution, a diagram of the sensitive layer for desirable sensitivity and the linear range was obtained, and the details are listed in Tables S1–S4.

For the microstructural template preparation in Step II, we 3D-printed (precision, 10 μm) the designed microdome-like structures on the resinous substrate according to the distribution diagram in Step I, and these structures served as a convex module to prepare the thermoplastic polyurethane (TPU) template since the concave mold tends to retain substances after film pouring, which not only is inconvenient to clean but also affects the microstructure shape. The TPU film was obtained after the inversion, and then the prepared MWCNT/PDMS conductive solution was poured and coated on the TPU film; finally, it was cured and peeled off the template. In Step III, an MWCNT/PDMS conductive film was sandwiched by the upper and lower flexible electrodes with watertight polyurethane (PU)-based adhesive tape^46^ for structure enhancement, which also endowed the fabricated sensors with humidity-insensitive properties (Fig. S2). Figure 2c shows the 3D surface scanning image of the printed sensitive microstructures (also illustrated in Fig. S3). Moreover, the fabricated films show multistage protrusions with different sensitivities on the scale of 500 μm (Fig. 2d), with a rationally predesigned distribution in both rows and columns.

### Sensing performance of the pressure sensor

**Fig. 3:**
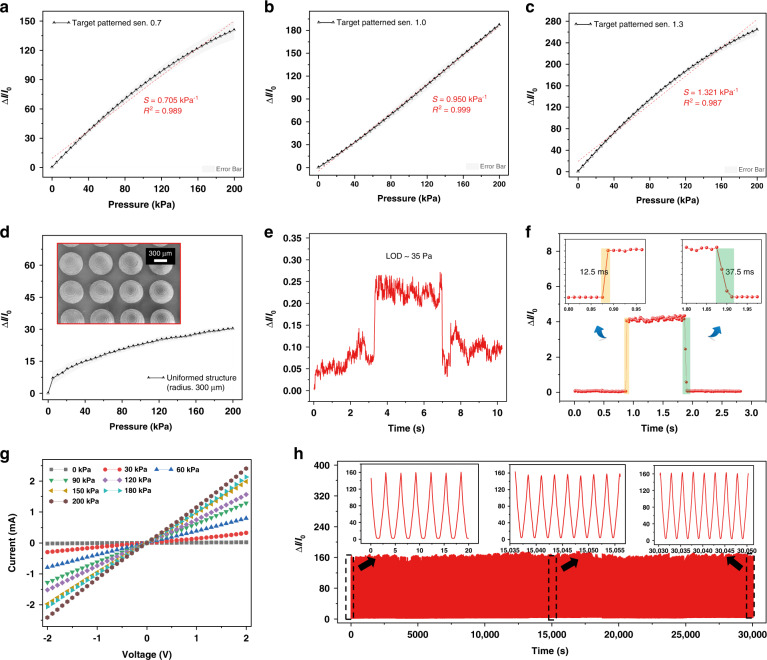
**a**–**c** Δ*I*/*I*_0_ versus the imposed pressure range of 0–200 kPa indicating the predesigned target sensitivity of 0.7, 1.0, and 1.3 kPa^−1^, respectively, based on the hyperelastic mechanism. **d** The sensitivity performance of the sensor with uniform microstructures with a radius of 300 μm. **e** Limit of detection. **f** The response/release time of the sensor. **g** Ohmic characteristics under the investigated voltage from −2 to 2 V. **h** The sensing reusability under 10,000 loading/unloading cycles

The as-fabricated microengineered pressure sensors prepared by the abovementioned procedure in the proposed proactive fashion featured target sensitivities varying from 0.7 to 1.3 kPa^−1^ over a working range of up to 200 kPa (Figs. S4–S7). Figure 3a–c shows the sensitivities of the pressure sensors fabricated via the proposed modified model with a broad linear working range from 0 to 200 kPa. Figure 3a shows that the measured sensitivity was 0.705 kPa^−1^, with superior linearity of 0.989 for a target sensitivity of 0.7, and the experimental deviation was only 0.7%, indicating that the sensitivity-tuned objective under a linear working region was achieved. Likewise, the predesigned sensors with target sensitivities of 1.0 and 1.3 kPa^−1^ (Fig. 3b, c) also showed expected results of 0.950 kPa^−1^ with a linearity of 0.999 and 1.321 kPa^−1^ with a linearity of 0.987, respectively. Notably, the maximum offset of the sensitivity arrangement (i.e., sensitivity error) was less than 5%, which further demonstrates the practical effectiveness of the positive design scheme. Figure 3d compares the prototype sensors featuring no hierarchical structures with previously developed sensors in terms of sensitivity and dynamic range. It was observed that the sensitivity gradually became saturated as the pressure increased since the uniform pixels with a radius of 300 μm tended to be flattened while lacking contact compensation.

The hierarchical structures generated by positively microengineered design increased the compressibility of the micropatterned active layer by adding air voids among the structures to decrease the modules^47,48^, which can easily and quickly induce energy conversion upon application of unloading and loading pressure, thus increasing the response speed and detection limit^49^. Here, the LOD and response/release time was also explored (Fig. 3e, f). The prototype sensor was capable of detecting a subtle pressure of 35 Pa on a contact area of 1 cm^2^, corresponding to a mass of 0.35 g. The fast response (12.5 ms) and recovery (37.5 ms) times were attributed to the graded microdomes and shortened the reactive periods by up to four orders of magnitude, thus indicating that the sensors could be suitable for most applications, such as low-frequency signal detection and monitoring. Significantly, the ineluctable viscoelastic behavior of hyperelastic materials can result in hysteresis of the relaxation time^17,50^, but the patterned structures could minimize this problem by decreasing the relative volumes without altering the sensing area.

To demonstrate the ohmic characteristics, the current–voltage (*I*–*V*) curve from −2 to 2 V was investigated under various pressures, and good linearity with a stable response was observed (Fig. 3g). In addition, as shown in Fig. 3h, the reusability of the sensor at a periodic pressure over 30,000 s was evaluated, and the insets emphatically show the good reusability and stability of the sensors even after 10,000 loading/unloading cycles with a consistent swift response. This favorable sensing performance is suitable for potential applications to better demonstrate the proposed positive design strategy with customizable sensitivity over highly linear regions.

### Theoretical validation of the hyperelastic and Hertzian models for the positive design strategy for comparison

**Fig. 4:**
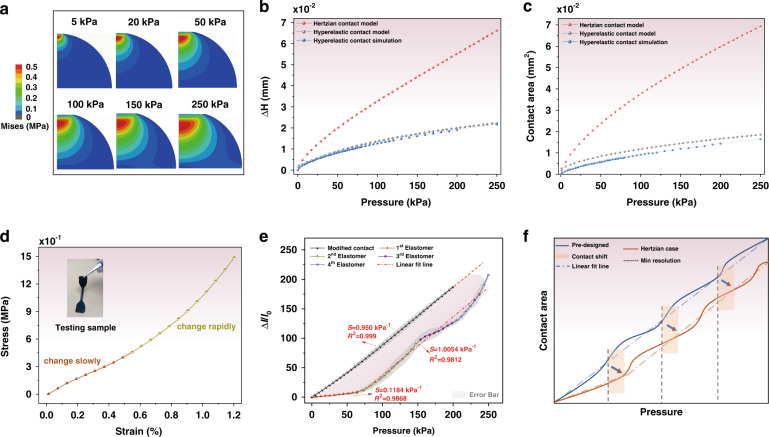
**a** FEA simulation results demonstrating the deformation conditions and Von Mises stress field of one hierarchical pixel with a radius of 200 μm. **b** The changing height and **c** contact area of a single hierarchical pixel in the pressure range of 0–250 kPa with the comparison of the proposed hyperelastic contact, Hertzian contact, and FEA methods. **d** The strain‒stress curve of the MWCNT/PDMS sensitive sample. **e** The anti-saturated compensation process as hierarchical structure compression. **f** The mechanism of the elastic contact shift

In addition to applying the developed modified hyperelastic model to sensor fabrication to realize the desired sensitivity and working range with high linearity, we validated the contact model by applying the FEA method to the investigation of the compression behavior of a single microstructural pixel. In addition, the Hertzian model has also been employed to describe the mechanism of hyperelastic compression. Figure 4a–c shows the height and area parameters determined with the hyperelastic contact, FEA, and Hertzian contact models for a single microstructural pixel versus an imposed pressure of up to 250 kPa.

The stress and deformation conditions over the dynamic compression process are shown in Fig. 4a, and the applied model and parameters are described in the Experimental Section. Figure 4b displays the theoretical results of the compression height change based on the hyperelastic model, the Hertzian contact model, and the corresponding FEA simulations. It was found that in the region of 0–250 kPa, the Hertzian height change increased at a certain rate without any sign of saturation, indicating that constant Young’s moduli existed during the whole loading process that did not conform to the intrinsic properties of hyperelastic materials. Our developed contact model corrected the above defects, and the calculated results were well correlated with the simulations. This can be further demonstrated with the contact area, as depicted in Fig. 4c. In summary, the PDMS substrate is a typical hyperelastic material whose Young’s modulus will increase with greater deformation in a nonlinear fashion (Fig. 4d).

To evaluate the linear sensitivity of the programable pressure sensor, Hertzian contact theory was then applied to the procedure of positive design (Text S2) in comparison with our proposed contact model. Figure 4e presents the Hertzian sensitivity performance over a predesigned working range (~200 kPa). We compared this performance with that of our prepared sensor with a sensitivity of 1.0 kPa^−1^ at the modified contact line based on hyperelastic mechanisms, and the actual output response curve of the Hertzian contact deviated considerably from the expectation for an elastic contact shift. Specifically, in the range of 0–60 kPa, the Hertzian contact sensitivity remained at only 0.1184 kPa^−1^, and the turning point (i.e., the point at which the former-staged structure is compressed to the top of the latter one) was also more delayed than the preset pressure, as Fig. 4f illustrates, from 30, 60, and 90 kPa to 70, 150, and 200 kPa, naturally extending the predesigned range from ~200 to ~250 kPa. It was suggested that the elastic contact shift occurred on the Hertz model with large deformations, which is detailed in Text S3.

### Application demonstration of the proposed sensor

**Fig. 5:**
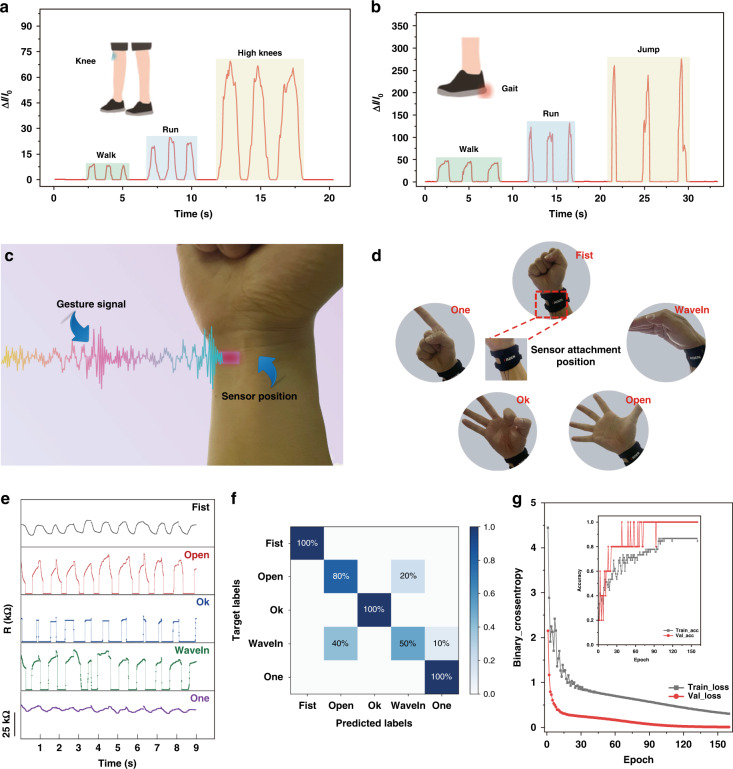
**a**, **b** The physiological signal changes of lower limb joints when performing different motions. **c** Sensor attachment position for gesture signal recognition. **d** Five testing gestures: fist, wave in, hand open, ok-sign, and one-sign. **e** Periodic resistance changes recorded by the hierarchical microstructure-based sensor as performing the testing gestures. **f** Confusion matrix for weight distinction derived from 10-fold machine learning with a prediction accuracy of >86.0%. **g** The binary cross entropy loss for assessing the effectiveness of the loss function, and the inset shows the prediction accuracy under fast mathematical convergence

Low-frequency physiological signals play a significant role in human‒machine interactions and medical rehabilitation, for example, in ambulatory activity recognition^51^ or hand gesture recognition^52^. Our prototype pressure sensor featuring superior flexibility was attached to several parts of subjects for real-time signal detection and recognition. Figure 5a, b shows the changes in the physiological signals of lower limb joints as the subjects performed different motions. Specifically, when the sensor was mounted on the knee of a volunteer (61 kg) performing walking, running, and high knee exercises (Fig. 5a), the quick changes in sensing resistance, with average readings of 7, 22, and 65, obviously reveal three actions corresponding to the above motions with distinct amplitudes, which can be employed to make a clear distinction. Similarly, to recognize various gaits, including jumping as the sensor was placed on an insole below the heel (Fig. 5b), which also demonstrates that the prototype sensor can exhibit a prompt response with instant electrical changes (approximately 50, 120, and 245, respectively) during knee or foot exercises over a large working region (~185 kPa). In addition, Fig. 5c shows that a sensor with customized sensitivity is available for detecting and distinguishing different hand gestures when attached to the pronator quadratus muscle, which helps to pronate whole wrist movements^53^. Afterward, we also applied our flexible sensor to identify hand motions, including *fist*, *wave in*, *hand opening*, *ok-sign*, and *one-sign* (Fig. 5d). Notably, the process of performing the predesigned gestures was conducted over ten times to verify the signal repeatability for identical actions, and Fig. 5e indicates that the actual signals were capable of reflecting the differences in muscular activities. For instance, the resistances captured during the performance of the one-sign gesture show significant peaks between 25.5 and 30.2 kΩ, with both rising and falling, indicating the implementation of current motion, such as from 1.5 to 2.5 s.

Machine learning combined with gestural pressure data has been proven to be an effective approach to identifying subjects’ intentions through specific hand movements^54–56^. Herein, we have adopted a three-layer back propagation (BP) neural network for gesture classification, and raw signals have been captured previously to further demonstrate the precise feedback that our proposed sensor could achieve. With the assistance of the signal preprocessing procedure, the raw data exhibited higher robustness via feature extraction, including the peak-to-peak value, mean value, root mean square, waveform factor and peak factor. We then employed the K-fold cross-validation method to evaluate our developed classification model by dividing the dataset into K categories, utilizing each of them as a test set and the others as a training set. In this work, the whole dataset was separately tested 10 times, and the average prediction accuracy was considered as the final performance evaluation in the confusion matrix, as shown in Fig. 5f. The results show that our pressure sensor integrated with the BP neural network has an overall recognition accuracy of >86.0% for all five gestures overall testing sessions, indicating classification performance via single-channel signals to achieve five-gesture recognition superior to that of other multichannel input methods in similar cases^57,58^. To access the effectiveness of the loss function in our classification model, we established the binary cross-entropy loss, as shown in Fig. 5g, which states that fast convergence can be achieved under less than 30 epochs in both the training and testing loss. The inset shows that the prediction accuracy can also complete mathematical convergence quickly (<90 epochs) while realizing impressive recognition performance. Notably, Fig. 5g reflects only the first session of the 10-fold validation. Therefore, our prototype sensor based on a positive strategy design can become a preferable selection for human‒machine interaction by means of machine learning.

## Conclusion

A positive design strategy using a hyperelastic contact theoretical model for developing customizable sensitivity- and linearity-tuned flexible piezoresistive pressure sensors based on microhierarchical dome-like structures has been proposed based on our newly modified contact theoretical model with hyperelastic mechanics. Three prototype samples were designed and fabricated, and they exhibited different sensitivities varying from 0.7 to 1.3 kPa^−1^ over a linear region of up to 200 kPa, featuring a sensitivity error <5% and linearity of ~0.99, consistent with expectations. The potential applications of the sensors in both ambulatory activity and hand gesture recognition were successfully demonstrated. Thus, our work highlights that sensors with the desired sensitivity and the required linear region can be calculated, predesigned, and fabricated via such a positive design scheme.

## Experimental section

### Preparation of a TPU solution and an MWCNT/PDMS conductive solution

The precursor solution of TPU was prepared by dissolving 20 wt% TPU (Bayer MaterialScience) in N,N-dimethylacetamide (DMAC, Sinopharm Chemical Reagent Co., Ltd.), and stirring at 50 °C for 12 h in complete dissolution. To prepare the MWCNT/PDMS solution, 1 wt% MWCNT powder was mixed into 5 mL of N-hexane (Aladdin), and ultrasonic irradiation was conducted at 53 kHz for 5 h. Then, 1 g of PDMS base (Slygard 184, DOW Corning, USA) and a curing agent in a weight ratio of 10:1 were poured into the mixed MWCNT/N-hexane solution and stirred at room temperature for 12 h.

### Fabrication of a pure TPU die from a 3D printed template

3D-printed templates with gradient architectures were adopted to prepare pure TPU dies for further manufacturing of the sensitive layer. The predesigned mold with microhierarchical structures of certain orders was fabricated by means of commercial high-precision equipment (S240, Boston Micro Fabrication Material Technology Inc., Shenzhen, China) and was characterized by projection microstereolithography (PμSL) 3D printing technology. After purification and UV curing of the printed convex module, the second mold with reverse-domed structures was obtained by casting the TPU solution onto the mold to replicate the customized microfeatures.

### Fabrication of an MWCNT/PDMS conductive film for the sensitive layer

For the secondary inversion to obtain the TPU film, the prepared 20 wt% TPU solution was poured onto the multistage hemispherical microstructure mold, kept at 100 °C for 1 h to cure, and then peeled off. Next, the prepared MWCNT/PDMS conductive solution was poured and coated on the TPU film and placed in a vacuum environment for 5 min so that the conductive solution could completely fill the micropits on the template. Then, it was kept at 80 °C for another 1 h, and the sensitive film was cured and peeled off from the template.

### Finite element analysis

FEA analysis was conducted to study the contact deformation between the upper electrodes and microhierarchical sensitive layers under various compression conditions. A two-dimensional model of protruding microstructures of a half single pixel in accordance with the equivalent strain relationship was built for simplicity. The microstructured composite conductive film and PU/Cu electrode as the rigid plates were modeled with isotopically elastic materials with Young’s moduli of 210 GPa and a polynomial hyperelastic model (the set parameters are listed in Table S5), respectively. In addition, frictionless contact on tangential behavior was also assumed as the contact interaction. The final compressed height and contact area were recorded as the pressure increased up to 250 kPa with the simulation method of direct iteration. The initial contact area was determined by the upper limit of the normalized height (i.e., 0.9) in our proposed modified hyperelastic contact model, and the corresponding pressure in terms of sensor size is detailed in Table S6.

### Characterization and measurements

The 3D surface morphology of the MWCNT/PDMS sensitive film was probed using a 3D laser scanning confocal microscope (VK-X1000, KEYENCE). The SEM images were obtained by means of a scanning electron microscope (SU-70, Hitachi, Japan). The external stimuli were induced by an electromechanical performance testing machine (E43.104, MTS Co., Ltd.), and the output resistances were recorded and measured via a digital multimeter (Agilent 34410A, KEYSIGHT). The output signals of the gesture recognition task were collected by our homemade FPGA-based collection board with the assistance of an ADC processor (AD7606, Liaoning Kangwei Technology Co., Ltd.).

### Prototype sensor test

The nominal height $\emptyset _c$ cannot be integrated at the pole position relative to the equator for whose area will be reduced to zero, which means providing the preload to create the initial contact area was the first necessary step for evaluating the sensing sensitivity. In this work, we preset the initial nominal height to 0.9, and the corresponding preloading pressure for each sensor of different sensitivities is listed in Table S6.

## Supplementary information

Supporting information-R1
