# Supplementary material for: A flexible pressure sensor with highly customizable sensitivity and linearity via positive design of microhierarchical structures with a hyperelastic model
Source: Microsyst Nanoeng. 2023 Jan 4;9:5. doi: 10.1038/s41378-022-00477-w (PMC9810721; doi:10.1038/s41378-022-00477-w)
Supplement: Supplementary file 1 — Supporting information-R1 [file 41378_2022_477_MOESM1_ESM.docx]

**Supporting Information**

Highly Customizable Sensitivity and Linearity Tuned Flexible Pressure Sensor via Positive Design of Micro-hierarchical Structures with Hyperelastic Model

*Zhenjin Xu^1, 2^, Dezhi Wu^1, 2,^ *, Zhiwen Chen^1, 2^, Zhongbao Wang^1, 2^, Cong Cao^1, 2^, Xiangyu Shao^1^, Gang Zhou^3^, Shaohua Zhang^3^, Lingyun Wang^1, 2,^ *, Daoheng Sun^1^*

^1^Department of Mechanical and Electrical Engineering, Xiamen University, Xiamen, 361005, China

^2^Shenzhen Research Institute of Xiamen University, Shenzhen, 518057, China

^3^Beijing Key Laboratory of Long-Life Technology of Precise Rotation and Transmission Mechanisms, Beijing Institute of Control Engineering, Beijing 100094, China

*Corresponding author

E-mail address: wdz@xmu.edu.cn (D. Wu), wangly@xmu.edu.cn (L. Wang)


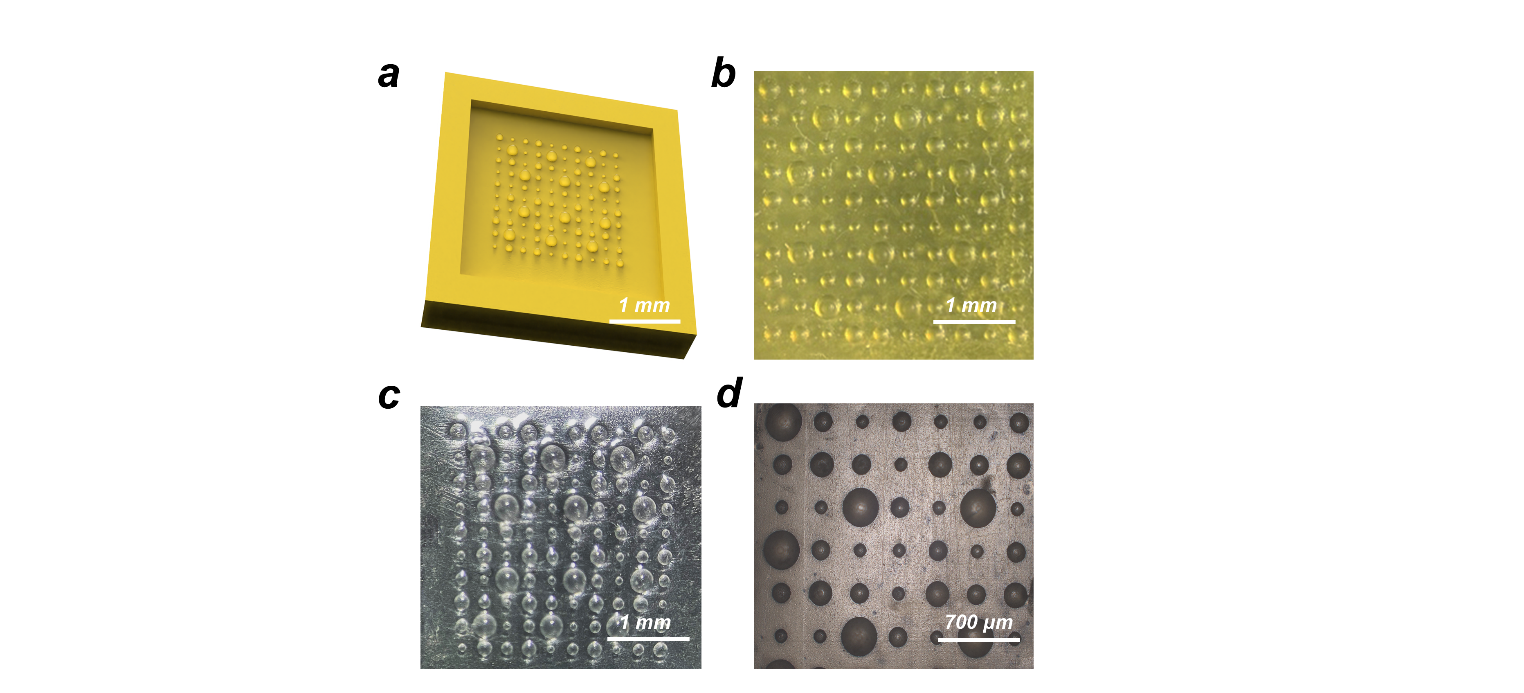


**Fig. S1** (a) Design diagram of the pre-designed template with the sensing sensitivity of 1.0 kPa^-1^ based on Hertzian contact. Optical images of (b) the 3D printed resinous template for the convex module, (c) the TPU concave mold, and (d) the fabricated sensitive layer with micro-hierarchical structures.


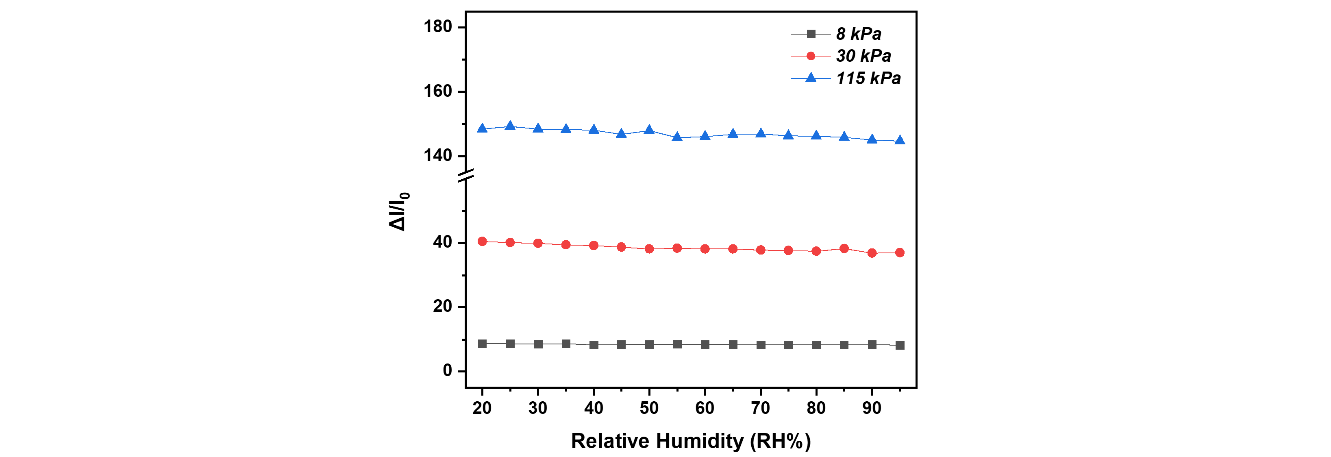


**Fig. S2** ΔI/I_0_ versus the relative humidity of 20-95 % RH indicating the proposed sensors with PU-based water isolating encapsulation not sensitive to humidity.


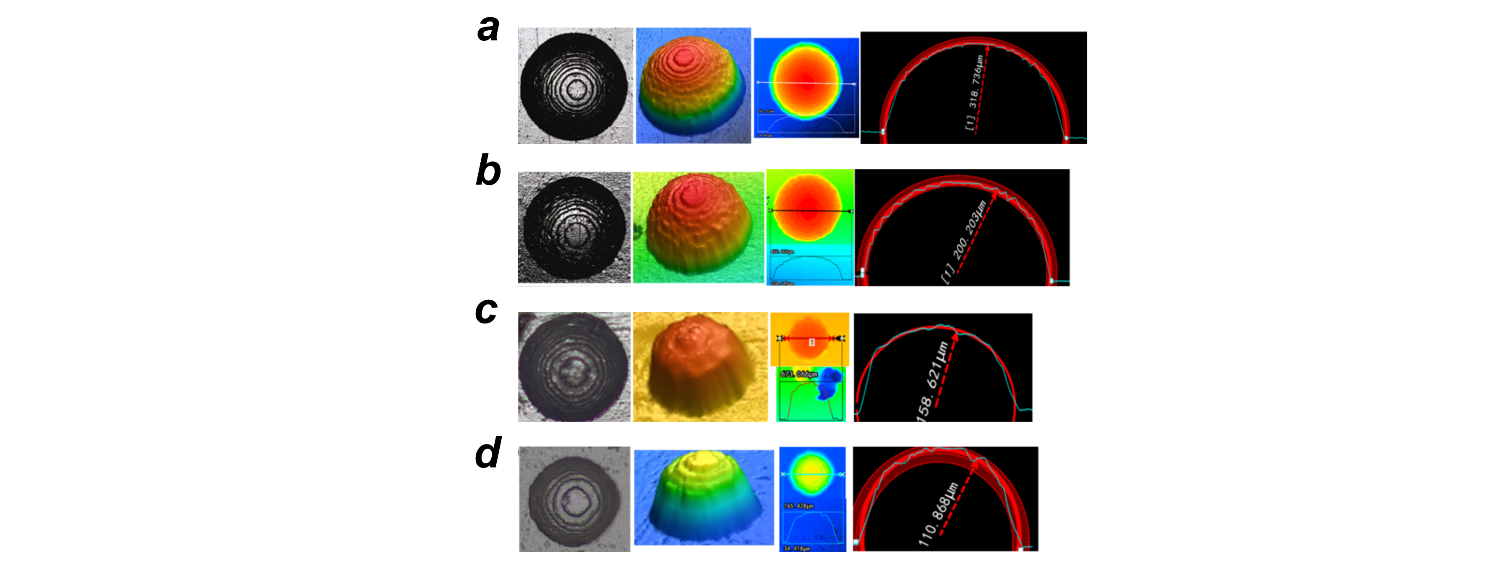


**Fig. S3** The microstructure measurement of the sensitive film via 3D surface scanning. (a) Stage I; (b) Stage II; (c) Stage III; (d) Stage IV.


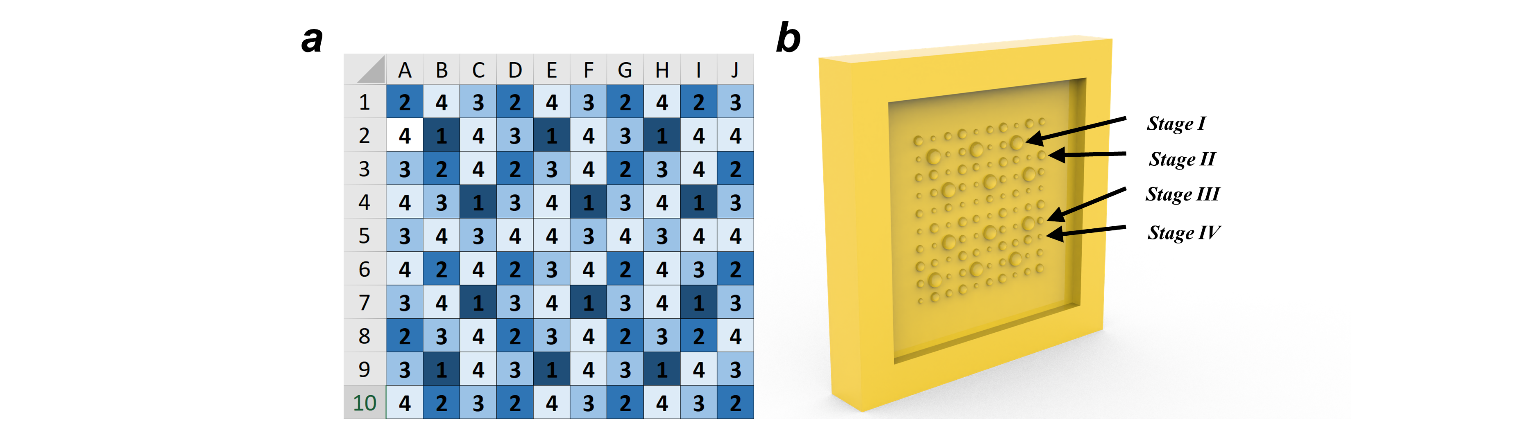


**Fig. S4** The hemispheric microstructures with the sensitivity of 1.0 kPa^-1^ based on Hertzian contact theory. (a) The distribution of microstructures. (b) The corresponding 3D printed diagram.


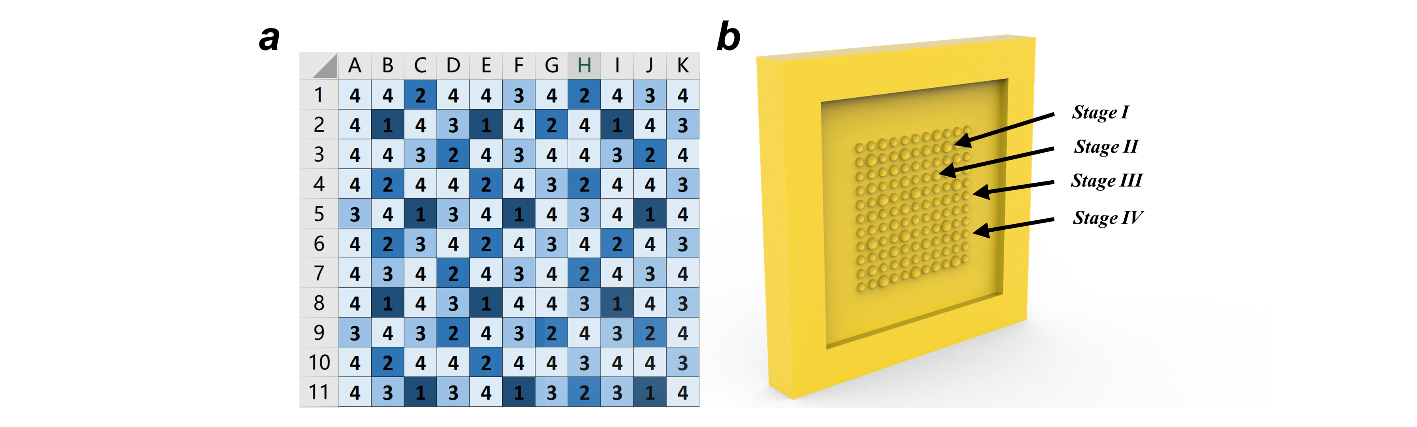


**Fig. S5** The hemispheric microstructures with the sensitivity of 0.7 kPa^-1^ based on our modified hyperelastic model. (a) The distribution of microstructures. (b) The corresponding 3D printed diagram.


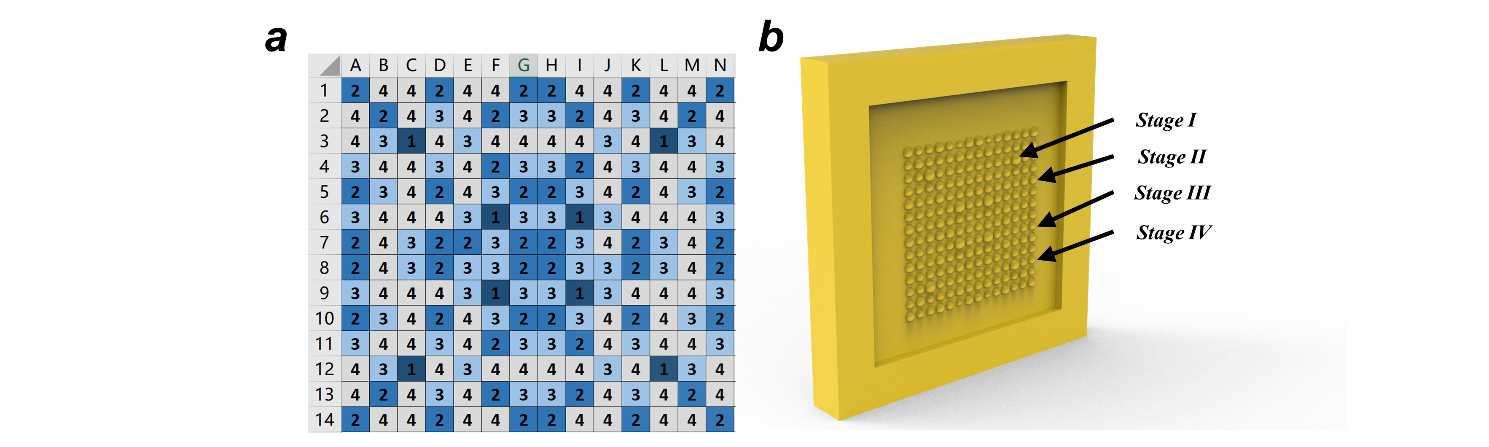


**Fig. S6** The hemispheric microstructures with the sensitivity of 1.0 kPa^-1^ based on our modified hyperelastic model. (a) The distribution of microstructures. (b) The corresponding 3D printed diagram.


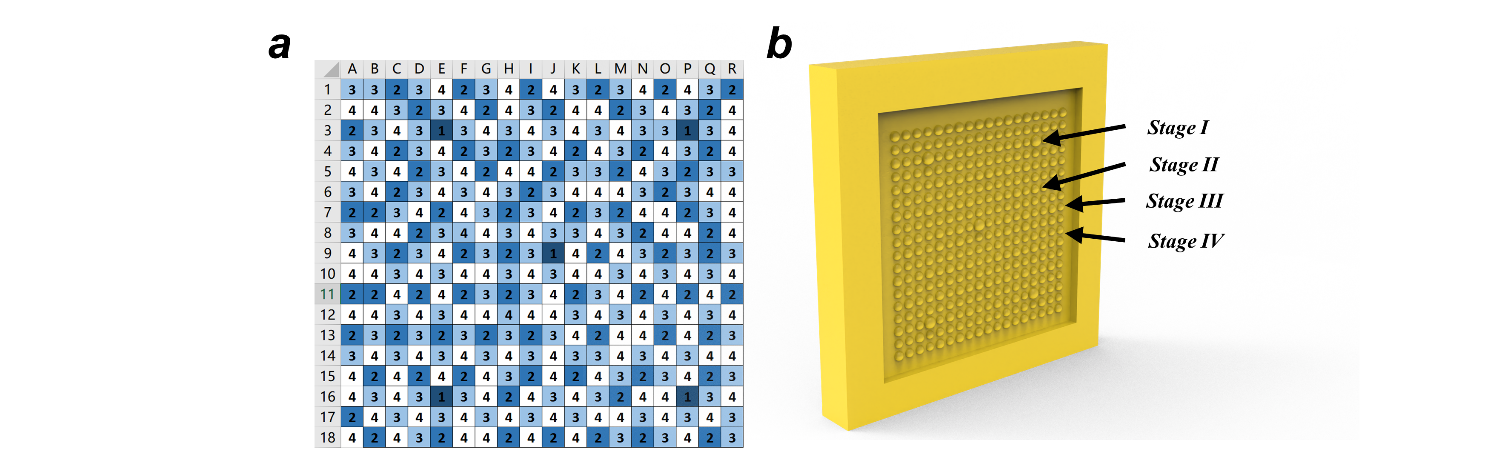


**Fig. S7** The hemispheric microstructures with the sensitivity of 1.3 kPa^-1^ based on our modified hyperelastic model. (a) The distribution of microstructures. (b) The corresponding 3D printed diagram.

**Table S1**. The size and its number of hemispheric microstructures with the sensitivity of 1.0 kPa^-1^ based on Hertzian contact theory

| Stages | 1 | 2 | 3 | 4 |
| --- | --- | --- | --- | --- |
| Radius (R /μm) | 300 | 190 | 150 | 100 |
| Number (m / #) | 12 | 20 | 32 | 36 |

**Table S2**. The size and its number of hemispheric microstructures with the sensitivity of 0.7 kPa^-1^ based on our modified hyperelastic model.

| Stages | 1 | 2 | 3 | 4 |
| --- | --- | --- | --- | --- |
| Radius (R /μm) | 300 | 260 | 230 | 220 |
| Number (m / #) | 12 | 19 | 31 | 59 |

**Table S3**. The size and its number of hemispheric microstructures with the sensitivity of 1.0 kPa^-1^ based on our modified hyperelastic model.

| Stages | 1 | 2 | 3 | 4 |
| --- | --- | --- | --- | --- |
| Radius (R /μm) | 300 | 250 | 230 | 220 |
| Number (m / #) | 8 | 49 | 58 | 81 |

**Table S4**. The size and its number of hemispheric microstructures with the sensitivity of 1.3 kPa^-1^ based on our modified hyperelastic model.

| Stages | 1 | 2 | 3 | 4 |
| --- | --- | --- | --- | --- |
| Radius (R /μm) | 300 | 240 | 220 | 210 |
| Number (m / #) | 5 | 76 | 112 | 121 |

**Table S5**. The controlled parameters of the simulated model based on polynomial hyperelastic contact (apply smoothing: 3).

| Nominal stress | Nominal strain | Nominal stress | Nominal strain |
| --- | --- | --- | --- |
| 939.6505034 | 0.012790256 | 587331.0566 | 0.667614153 |
| 61842.58244 | 0.072378006 | 660358.4506 | 0.727081804 |
| 118622.2515 | 0.131878569 | 736104.0874 | 0.786661747 |
| 164954.9874 | 0.191431448 | 818325.6604 | 0.846166252 |
| 210063.2543 | 0.250895175 | 907581.5569 | 0.905713244 |
| 251581.1541 | 0.310471304 | 1006051.194 | 0.965173134 |
| 296258.4116 | 0.369971862 | 1112191.352 | 1.024753121 |
| 343998.8331 | 0.429522823 | 1229760.322 | 1.084259489 |
| 397666.9095 | 0.488986527 | 1352031.014 | 1.14380657 |
| 455397.3675 | 0.548570417 | 1486339.136 | 1.20327808 |
| 520489.1617 | 0.608070975 |  |  |

**Table S6**. The preloading conditions of 5 developed sensors with different sensitivities for reaching the nominal height of 0.9. Note: Hertz stands for whose sensor based on Hertzian contact, while Hyper means that based on our hyperelastic contact.

| Sensitivity | 1.0  (Hertz) | 0.7 (Hyper) | 1.0 (Hyper) | 1.3 (Hyper) | SAME (Hyper) |
| --- | --- | --- | --- | --- | --- |
| Sensor size (mm) | 10*10 | 9*9 | 10*10 | 11*11 | 10*10 |
| Initial force (N) | 0.014 | 0.014 | 0.0095 | 0.0059 | 0.168 |

**Text S1.** Modified contact model with hyperelastic mechanics

To systematically explain the compressing principle of the hemisphere-shaped structures on the sensor performance, the nonlinear contact can be modeled by hyperelastic mechanics, since PDMS is often considered as a homogeneous rubbery material that can present volume preserving as pressure imposes ^1^. When the force uniaxially applies to the hemisphere pole, the lateral expansion will be occurred in a nonlinear fashion (i.e., change in the height and radius). In our work, the neo-Hookean model, a common hyperelastic material model for large deformations (more than 20 %), is taken to seek for the analytic solution of compressive conditions of the proposed hemisphere-shaped structure which inspired by Rodriguez et al and Guido van Schie et al, trying to figure out the nonlinear relationship between internal strain and stress ^2,3^. Specifically, for an incompressible neo-Hookean material ^4^, the strain energy density function can be calculated by

|  | $W =C_{1}(I_{1} - 3)$ | (1) |
| --- | --- | --- |

where $C_{1}$, and $I_{1}$ represent the material constant and the first invariant of right Cauchy-Green deformation tensor, respectively. Given in terms of the principal extension ratios $\lambda_{1}$, $\lambda_{2}$, and $\lambda_{3}$, $I_{1}$ is obtained from

|  | $I_{1} =\lambda_{1}^{2}+\lambda_{2}^{2}+\lambda_{3}^{2}$ | (2) |
| --- | --- | --- |

The resulting stress derived by principle stretches is referred to as Cauchy stress tensor, and only the subcomponents $\sigma_{ii}$ of the stress tensor in the three corresponding directions (i.e., *i* = 1, 2, 3) promote the change state inside the hemisphere since there is no circumferential constraints. For a localized contact point, employing Equation we can rewrite (1),

|  | $\sigma_{11}-\sigma_{33} =\lambda_{1}\frac{\partial W}{\partial\lambda_{1}}-\lambda_{3}\frac{\partial W}{\partial\lambda_{3}}$  $\sigma_{22}-\sigma_{33} =\lambda_{2}\frac{\partial W}{\partial\lambda_{2}}-\lambda_{3}\frac{\partial W}{\partial\lambda_{3}}$ | (3) |
| --- | --- | --- |

We can note that for an incompressible neo-Hookean model, with

|  | $\lambda_{1}{\cdot\lambda}_{2}{\cdot\lambda}_{3}=1$ | (4) |
| --- | --- | --- |

we get

|  | ${\frac{\partial W}{\partial\lambda_{i}}=2C_{1}\lambda}_{i} (i=1, 2, 3)$ | (5) |
| --- | --- | --- |

By combining with Equation (1)(2)(5), we would have for Equation (3):

|  | $\sigma_{11}-\sigma_{33} =2C_{1}(\lambda_{1}^{2}-\lambda_{3}^{2})$  $\sigma_{22}-\sigma_{33} =2C_{1}(\lambda_{2}^{2}-\lambda_{3}^{2})$ | (6) |
| --- | --- | --- |

Now, we once again revisit the constraint conditions in our case of uniaxial compression, the boundary condition for the stress are as follows: the normal stress only exits at sphere surface within the contact area $\sigma_{33}$ while at elsewhere are zero, that is

|  | $\sigma_{11}=\sigma_{22} =0$  $\lambda_{1}= \lambda_{2}=\lambda$  $\lambda_{3}= \frac{1}{\lambda_{2}}$ | (7) |
| --- | --- | --- |

Therefore, Equation (6) can be displayed with Equation (3.2) by

|  | $\sigma_{33}=2C_{1}(\frac{1}{\lambda^{4}}-\lambda^{2})$ | (8) |
| --- | --- | --- |

or it can be written as

|  | $\sigma=\frac{f}{A}=2C_{1}(\alpha^{-2}-\alpha)$ | (9) |
| --- | --- | --- |

with

|  | $\alpha=\lambda^{2}$ | (10) |
| --- | --- | --- |

For better describing the compressing process of the hyperelastic hemisphere architectures, the spherical neo-Hookean model is initially employed for this case following by Equation (10). Furthermore, consider the entire sphere would be sliced into infinitely many layers along the latitude, and the equatorial one now can be regarded as a cylinder based on Calculus method of radius *R^*^* and thickness *(dz^*^)_e_*. According to Equation (9), the compressive stress of equatorial undeformed layer can be defined by

|  | $\sigma_{e}=\frac{f}{\pi{R^{*}}^{2}}$ | (11) |
| --- | --- | --- |

Clearly, the external force will transmit through every layer without any loss, and then the geometric change at equator as a strain ratio of *α_e_*, as shown in **Fig. S**8 that is

|  | ${(dz)}_{e}=\alpha_{e}{({dz}^{*})}_{e}$ | (12) |
| --- | --- | --- |


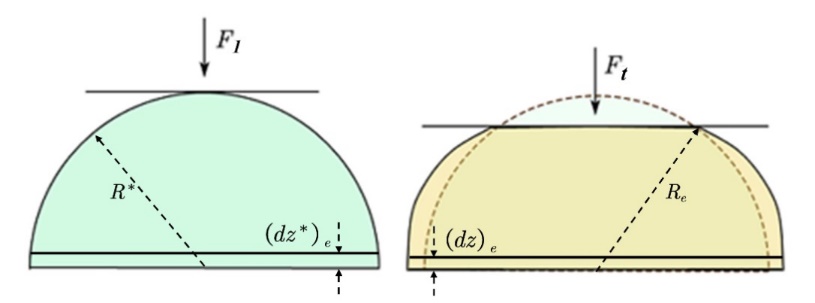


**Fig. S8** The strain ratio of the equator.

Meanwhile, such a layer holds its volume constantly because of its incompressibility:

|  | $\pi{R^{*}}^{2}\cdot\left( {dz}^{*} \right)_{e}=\pi{(R_{e})}^{2}\cdot{(dz)}_{e}$ | (13) |
| --- | --- | --- |

with Equation (13), the strain ratio at equator *α_e_* can be obtained as

|  | $\alpha_{e}={(\frac{R^{*}}{R_{e}})}^{2}$ | (14) |
| --- | --- | --- |

Next, we consider any one layer of the material with the same thickness of that of the equator at an arbitrary vertical distance *y^∗^* from the sphere center (**Fig. S**9). By taking the radius of this designated layer (*x^∗^*), we have

|  | $y^{*2}=R^{*2}-x^{*2}$ | (15) |
| --- | --- | --- |


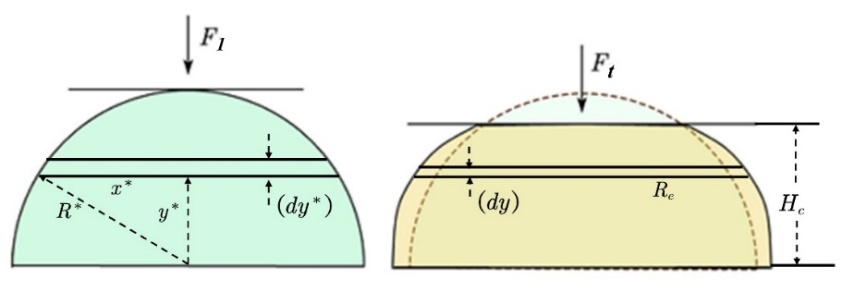


**Fig. S9** The strain ratio of the equator.

We now need to develop the expression for the compressed radius as *R_c_*, that is

|  | $H_{c} =\int_{0}^{h_{max}} dy=\int_{0}^{R_{*}} \alpha\cdot{dy}^{*}$ | (16) |
| --- | --- | --- |

where *α* represents the local strain ratio. Since the hypothetical rigid plate provides the interaction force to support the sphere, the deformation out of the imposed force will stay identical from the top to the bottom. With that in mind, only the either side of the sphere also follows the deduced deformation pattern, which is in accordance with our proposed hemisphere microstructure. So normalizing the dimensional form with respect to the vertical position, we can see that

|  | $\beta=\int_{0}^{1} \alpha_{\phi}d\phi$ | (17) |
| --- | --- | --- |

where

|  | $\beta=\frac{R_{c}}{R^{*}} and \phi= \frac{y^{*}}{R^{*}}$ | (18) |
| --- | --- | --- |

The hyperelastic material constant *C_1_* can be calculated by the second Lamé parameters ^5^ (i.e., shear modulus G), and then we further define the strain ratios to be dimensionless to Q, referring to Equation (9):

|  | $Q_{e} =\frac{\sigma_{e}}{G}$ | (19) |
| --- | --- | --- |

where *Q_e_* stands for (*α*^-2^ - *α*), so we could therefore figure out *α_e_* under certain imposed

pressure with Equation (19).

Now, for localized stress at any latitude to *ϕ* we can get

|  | $\sigma_{\phi} =\frac{f}{\pi{x^{*}}^{2}}={G(\alpha^{-2}- \alpha)}_{\phi}$ | (20) |
| --- | --- | --- |

where G represents the shear module ($G=\frac{E}{2(1+\mu)}$). Combined with the tensile test results (**Fig. 4**d), the Young's modulus of the material also increases with the increase of strain. During the compression process of the hemisphere, the stress is mainly concentrated on its top. When the external pressure is 250 kPa, the stress at "*∅* = 0.95" can reach 2.5 MPa, the corresponding Young's modulus is about 7 MPa, and its initial state is 0.8 MPa. Therefore, when calculating the strain during hemispherical compression, the Young's modulus is set as a variable varying with pressure, and its value gradually increases from 0.8 MPa to 7 MPa.

Inspired by the Equation (20), the change rate of nominal strain relative to the equator under compression at any nominal height can be calculated as follows:

|  | $\frac{\sigma_{\phi}}{G}={(\alpha^{-2}- \alpha)}_{\phi}$ | (21) |
| --- | --- | --- |

At this point, the strain ratio at the nominal height of the contact area *∅_c_* can also be figured out. Combining Equation (20) with (14) yields

|  | $\text{R}_{\text{c}}\text{ }\text{=}\text{ }\text{R}^{\text{*}}\text{×}\sqrt{\frac{\text{1-}{\text{∅}_{\text{c}}}^{\text{2}}}{\text{α}_{\text{c}}}}$ | (22) |
| --- | --- | --- |

where *R_c_* is the contact radius. Further by introducing Equation (17), the actual height change of the current compressive state can be expressed as

|  | $\text{∆h}\text{ }\text{=}\text{ }\left( \text{1-β} \right)\cdot\text{R}^{\text{*}}$ | (23) |
| --- | --- | --- |

As for the distribution of the microstructure array, we have discussed as the **Section 2.2**.

**Text S2**. The design scheme of microstructure array based on Hertzian contact theory

Hertzian contact is usually used to calculate the contact stress of nodes in the process of gear meshing, and it can also be used to calculate the sphere to sphere contact under small contact deformation. As applied the pressure with *f* combining the diameter of the sphere is *R_1_* and *R_2_*, respectively, the contact radius between them can be calculated by

|  | $C=\sqrt[\text{3}]{\frac{\text{3F}}{\text{4}}\text{×}\frac{\frac{\text{1-}{\text{μ}_{\text{1}}}^{\text{2}}}{\text{E}_{\text{1}}}\text{+}\frac{\text{1-}{\text{μ}_{\text{2}}}^{\text{2}}}{\text{E}_{\text{2}}}}{\frac{\text{1}}{\text{R}_{\text{1}}}\text{+}\frac{\text{1}}{\text{R}_{\text{2}}}}}$ | (24) |
| --- | --- | --- |

where *E_1_* and *E_2_* are the corresponding elastic modulus respectively, whereas *μ_1_* and *μ_2_* are the Poisson's ratio of them, respectively.

When the contact model specifically refers to the contact between the copper foil plane and the microstructural sphere of PDMS conductive composite, then *R_2_* reaches infinite, while the Young's modulus of copper foil is much greater than PDMS. At this time, Equation (23) can be simplified as:

|  | $C=\sqrt[\text{3}]{\frac{\text{3F}}{\text{4}}\text{×}\frac{\text{1-}\text{μ}^{\text{2}}}{\text{E}}\text{×R}}$ | (25) |
| --- | --- | --- |

where *R*, *E* and *μ* represent the radius of curvature, modulus of elasticity and Poisson's ratio of conductive composite microsphere. We have set E = 0.8 MPa, R = 200 μm, μ = 0.3.

Since the contact area can be expressed as $\text{S = }\text{π}c^{2}$, we can see that the compression height:

|  | $\text{∆h}\text{ }=\text{R-}\sqrt{\text{R}^{\text{2}}\text{-}\text{c}^{\text{2}}}$ | (26) |
| --- | --- | --- |

**Text S3**. The elastic contact shift happens on Hertz model with large deformations

The Young's modulus should remain unchanged when calculated by Hertz model, whose contact radius $R_{c}$ can be expressed as

|  | $R_{c}=\sqrt[\text{3}]{\frac{\text{3F}}{\text{4}}\text{×}\frac{\text{1-}\text{μ}^{\text{2}}}{\text{E}}\text{×R}}$ | (27) |
| --- | --- | --- |

where *R*, *E* and *μ* respectively represent the radius, elastic modulus and Poisson's ratio of conductive microstructure hemispheres under a rigid plate-sphere contact (**Text S**2). The involved parameters will keep fixed under ongoing compression, indicating that the actual deformation tends to be less than that calculated by Hertz model, which is the major cause for the deviation of output curve and the delay of turning pressure point. This better illustrates that Hertzian contact stress merely referring to slight deformation since the elastic properties should be supposed to keep constant under external stimuli ^6^. Although the deviation inevitably happens under imposed loads because of the elastic contact shift over large deformation, the gradient compensation for anti-saturation still works with our hierarchical structures from 2^nd^ to 4^th^ stages. Therefore, taken the dynamic characteristics and shape hierarchy of hyperelastic substrate for sensitive layer into account is to be an onramp for addressing the contact shift issue.

**Supplementary Reference**

1 Lee, H. M. *et al.* Modeling and application of anisotropic hyperelasticity of PDMS polymers with surface patterns obtained by additive manufacturing technology. *Journal of the Mechanical Behavior of Biomedical Materials* **118**, 104412, (2021).

2 Rodriguez, F., Patel, S. K. & Cohen, C. Measuring the modulus of a sphere by squeezing between parallel plates. *Journal of Applied Polymer Science* **40**, 285-295, (1990).

3 van Schie, G. *et al.* Correlating locations in ipsilateral breast tomosynthesis views using an analytical hemispherical compression model. *Physics in Medicine and Biology* **56**, 4715-4730, (2011).

4 Hackett, R. M. *Hyperelasticity Primer*. 2 edn, (Springer, Cham, 2018).

5 Slaughter, W. S. *The linearized theory of elasticity*. (Springer Science & Business Media, 2002).

6 Hertz, H. *Miscellaneous Papers, Chapter V: On the contact of elastic solids*. 146-162 (1896).
